# Picture This: A Review of Research Relating to Narrative Processing by Moving Image Versus Language

**DOI:** 10.3389/fpsyg.2019.01161

**Published:** 2019-06-26

**Authors:** Elspeth Jajdelska, Miranda Anderson, Christopher Butler, Nigel Fabb, Elizabeth Finnigan, Ian Garwood, Stephen Kelly, Wendy Kirk, Karin Kukkonen, Sinead Mullally, Stephan Schwan

**Affiliations:** ^1^ English, University of Strathclyde, Glasgow, United Kingdom; ^2^ Philosophy, University of Stirling, Stirling, United Kingdom; ^3^ Department of Clinical Neuroscience, University of Oxford, Oxford, United Kingdom; ^4^ English, Southern Regional College of Northern Ireland, Armagh, United Kingdom; ^5^ Film and Television Studies, University of Glasgow, Glasgow, United Kingdom; ^6^ Psychology, University of Strathclyde, Glasgow, United Kingdom; ^7^ Glasgow Women’s Library, Glasgow, United Kingdom; ^8^ Comparative Literature, University of Oslo, Oslo, Norway; ^9^ Neuropsychology, Newcastle University, Newcastle upon Tyne, United Kingdom; ^10^ Psychology, Leibniz-Institut für Wissensmedien, Tübingen, Germany

**Keywords:** narrative, media, reading, film, fiction, comprehension, literature, cognitive humanities

## Abstract

Reading fiction for pleasure is robustly correlated with improved cognitive attainment and other benefits. It is also in decline among young people in developed nations, in part because of competition from moving image fiction. We review existing research on the differences between reading or hearing verbal fiction and watching moving image fiction, as well as looking more broadly at research on image or text interactions and visual versus verbal processing. We conclude that verbal narrative generates more diverse responses than moving image narrative. We note that reading and viewing narrative are different tasks, with different cognitive loads. Viewing moving image narrative mostly involves visual processing with some working memory engagement, whereas reading narrative involves verbal processing, visual imagery, and personal memory (Xu et al., 2005). Attempts to compare the two by creating equivalent stimuli and task demands face a number of challenges. We discuss the difficulties of such comparative approaches. We then investigate the possibility of identifying lower level processing mechanisms that might distinguish cognition of the two media and propose internal scene construction and working memory as foci for future research. Although many of the sources we draw on concentrate on English-speaking participants in European or North American settings, we also cover material relating to speakers of Dutch, German, Hebrew, and Japanese in their respective countries, and studies of a remote Turkish mountain community.

## Introduction

Reading, and reading fiction in particular, for enjoyment has been positively correlated with young people’s attainment in a wide range of studies across different countries, many, such as the OECD’s PISA studies, involving large cohorts (Cunningham and Stanovich, 1998; PIRL, 2001, 2006; Organisation for Economic Co-operation and Development (OECD), 2002, 2010; Verghese et al., 2003; Mar et al., 2009; Hughes et al., 2010; Sullivan and Brown, 2013; McGeown et al., 2015; Ritchie et al., 2015; Sikora et al., 2019). Moving image narrative is experienced as easier than written narrative both to process and access (Salomon, 1979, 1984; Salomon and Leigh, 1984; Beentjes and van der Voort, 1988, p. 393–4; Ennemoser and Schneider, 2007; Jensen et al., 2016). But there is no evidence that watching fiction films for enjoyment confers comparable benefits in attainment. Since the advent of television, and then laptops, tablets, and smart phones, reading for enjoyment has faced ever more competition from the moving image (Merga, 2016, 2017; Merga and Roni, 2017). This is causing concern among policy makers and educationalists, among others (Scottish Government, 2007; Scholastic, 2015).

It is not clear exactly why reading narrative for enjoyment is associated with attainment. An analysis of data on identical twins by Ritchie et al. suggests that an association between reading and either genetic traits or socio-economic background cannot explain all of the benefits of reading for pleasure (Ritchie et al., 2015; see also Organisation for Economic Co-operation and Development (OECD), 2002). Nor is it clear whether, for example, watching highly crafted moving image fiction for enjoyment could have comparable benefits. A clearer understanding of the underlying mechanism of the effect could help to make the case for encouraging young people to enjoy fiction and guide policy. In this review, we first examine evidence on the similarities and differences between narrative processing across these media. There are substantial structural and experiential overlaps between narratives in media. But our review suggests that moving image narrative produces superior performance in the recall of sensory detail and information; readers have more divergent experiences than viewers; and variation between individuals affects the experience of verbal narrative more than that of moving image narrative. However, findings are hard to compare. Researchers use varied methods in the attempt to produce comparable stimuli in the two media, and the body of research is often fragmented and inconclusive.

In the second part, we explore approaches for future research. We suggest a change of focus from attempting to create equivalent stimuli. Instead, it may be fruitful to compare how early visual and verbal processing interacts with global and temporally extended experiences of narrative. In particular, we consider internal scene construction, supported by the episodic memory system, and the potential for differential engagements of verbal and visual working memory in different media. These mechanisms may be implemented in the ways that both lower and higher level causal patterns in narrative are constructed, through flexibility of concept instantiation. Fiction reading may draw more extensively on personal memory than watching moving image narrative and may involve more complex causal inferences.

Throughout the review, we use the term “story” for a minimal narrative structure underlying a narrative experience. We use “narrative” for stories rendered in words and/or images; stimuli extended in time and depicting at least one agent-like entity initiating and/or experiencing change in their environment (Gennette, 1980; Fludernik, 2008). “Moving image” refers to all kinds of screen-based moving image stimuli, from those seen in cinemas to those watched on phones. This term covers both animations and live action films. Most moving image narratives today include speech and other sounds. We follow many of the researchers discussed below and classify these as “moving image” but explore the question of hybridity in movies, the fact that they often combine speech, music, and either live action or animated images in a single narrative, where possible. “Verbal” refers to any language-based stimuli, spoken or written (or potentially signed, though we have no examples of signed narratives). “Text” refers to verbal stimuli organized at the level of discourse (rather than, for example, letter, word, or single clause), whether spoken or written. We use “transportation” to describe a mode of experiencing narration, more or less independent of medium. This is distinct from “immersion” or “presence,” terms used to describe the experience of a medium, more or less independent of narration (Busselle and Bilandzic, 2008).

## Similarities in Narrative Processing Between Text and Moving Image

Our understanding of similarities is informed by a model of narrative processing developed in the 1980s and 1990s, initially for text. This model combines “situation modeling” of a narrative with “event segmentation.” Situation modeling emerged from a range of earlier work focused on narrative processing as an information-focused task relating to real-life schemas (Meyer, 1975; Rumelhart, 1975, 1977; Mandler and Johnson, 1977; Thorndyke, 1977; Poulsen et al., 1979; Stein and Glenn, 1979; Lichtenstein and Brewer, 1980). Event indexing also emerged from work on real-life processing (Newton, 1973; Zacks and Tversky, 2001). Using this model, a range of work by Zacks and colleagues suggests that in real life, verbal narrative and moving image narrative, we attend to the same dimensions (characters’ goals, their relationships with objects, and their location) using the same mechanisms of heightened attention to event boundaries at a range of timescales (Speer et al. 2007a; Zacks et al., 2009; Zacks, 2015, p. 29).

Situation modeling also provides evidence for distinctions made by narratologists, anthropologists, and folklorists between a minimal structure that can be reused (story) and the experience of a particular iteration of such a structure (narrative) (Aarne and Thompson, 1961; Propp, 1968; Genette, 1980; Shklovsky, 2011, p. 25, 31). The minimal structure consists of a sequence of events and relates to modeling and updating the narrative across dimensions, such as changes in characters’ goals (Zwaan and Radvansky, 1998). The model generates bounded episodes in a sequence through event perception which segments time into hierarchically organized events, by directing attention to points when several dimensions change at once.

There has been less empirical work on the experiential aspects of narrative, though here too there is evidence of a shared narrative processing across media. Investigating the potential for narrative to improve abilities to empathise, Kidd and Castano (2013), for example, found that literary fiction, as distinct from popular genre fiction, produced improved performance in theory of mind tests, while Black and Barnes (2015) found the same for viewing award-winning, as opposed to less critically acclaimed, television drama (see also Mar et al., 2009; Koopman and Hakemulder, 2015). Hakemulder similarly finds that both films and texts which carefully craft form, the *way* the story is told, produce a richer experience of meaning (Hakemulder, 2007). Both media are capable of supporting transportation, a narrative state involving “imagery, emotional response and attentional focus” (Green and Brock, 2000; Green et al., 2008; Bal and Veltkamp, 2013).

It is not surprising then that theoretical and empirical work in both psychology and humanities reveals substantial overlaps in the structures and affordances of the two media, with both requiring readers, hearers, and viewers to learn or understand *ways* of representing material. Magliano et al. show how “Mapping processes” between shots, for example, are akin to the “bridging inferences we know that readers generate” in reading, as demonstrated by psycholinguistic research (Magliano et al., 2013, p. 79). Shot sequences of faces and postures can enable inferences about characters’ inner states, which authors supply through, for example, narrative voice or free indirect speech, and through direct information about the inner states or indirect information about facial expressions and stance (Magliano et al., 2013, p. 80).

Aesthetic approaches to form also suggest continuity between narrative media. Hakemulder defines “literariness” as deviation from the norms of representation and finds that it can enhance both film and text narratives (Hakemulder, 2004, 2007). Simpson suggests cinematic analogues for stylistic textual features that create a sense of urgency (Simpson, 2014). Kraft compares manipulations of camera angle to variation in verbal narrators’ points of view (Kraft, 1987). Forceville makes a systematic comparison between Ian McEwan’s novel *The Comfort of Strangers* and Harold Pinter’s film adaptation to show how visual metaphors, cross cuts, and ambiguous “point of view shots” can replicate effects generated by McEwan using verbal style (McEwan, 1981; Pinter, 1990; Forceville, 2002). Lang et al. suggest that audiovisual narratives can share some of the structure of oral conversational narratives (Lang et al., 1995). Work in the humanities on “transmedia” narrative theory has also identified both affordances specific to particular media, and ways in which these can create formal equivalence (Ryan, 2004; Walsh, 2006; Kukkonen, 2011, 2013).

But both media seem to depend on similar kinds of medium-specific learning, rather than just carrying over generic knowledge into an understanding of what is being represented. For example, Schwan and Ildirar worked with adult first-time viewers of television to explore what formal processes they needed to comprehend moving image narrative. These viewers found some aspects of continuity across shots straightforward, but not all. For example, they interpreted changes in camera position across shots as changes in the position of the person filmed, and had to learn to see film as a crafted medium creating “a coherent whole” (Schwan and Ildirar, 2010; Ildirar and Schwan, 2015). Viewers of dynamic scenes similarly learn to combine different views which may maximize relevant information rather than being organized around real-life experience (Friedman and Waller, 2008; Garsoffky et al., 2009; Huff and Schwan, 2012). Film viewers must also learn to identify event boundaries, which are missing from shots or cross cuts (Schwan et al., 2000; Schwan and Garsoffky, 2004; Shimamura et al., 2014, 2015; Smith and Mital, 2015). In the same way, learning to understand spoken language and/or decode print is not sufficient to understand verbal narrative. Written discourse comprehension involves an ability to conceptualize a narrator who, unlike a face to face interlocutor, has only a notional location in time and space, and to correctly interpret deixis (terms relating to speaker location, such as “this” versus “that”) in relation to that notional location (Jajdelska, 2007). Without these skills, even children who are skilled at decoding written words and sentences (converting them into speech) cannot always draw appropriate inferences from narrative text (Yuill and Oakhill, 1991).

Both media then share structures of comprehension through situation modeling and the marking of event boundaries. Both can support transportation and enhanced empathy. Both require their audience to appreciate their status as artifacts, and both can vary the ways in which information is conveyed for comparable, if not equivalent, esthetic effects.

## Differences in Narrative Processing by Text and Moving Image

Baggett compared film and text narrative processing in a series of experiments (Baggett, 1975, 1979, 1984; Baggett, 1981; Baggett and Ehrenfeucht, 1982) and characterized by careful and painstaking efforts to create equivalent stimuli across the two media. These included using a popular children’s film with no dialogue (“The Red Balloon”) to generate a text narrative which subjects judged had exactly the same episodes (1979). These included taking a popular children’s film which had no dialogue (“The Red Balloon”), creating a text version of the narrative, and then inviting raters to confirm that the text and the film had the same number of episodes. The approach to generating materials drew on theories of narrative structure (Kintsch and Van Dyke, 1975) comparable to the later situation model and event boundary approach. Baggett and Ehrenfeucht later looked at content rather than episodic structure, using 180 stills and 20 free recall protocols from viewers to rewrite the written source for a 1953 animated film. They used this to create an audio text of exactly the same length as the film and using the same dialogue (Baggett and Ehrenfeucht, 1982).

Baggett’s findings on structure support the work on event indexing and situation modeling discussed above; both readers and viewers segmented the story in similar ways. In cued recall after 7 days, however, readers were more likely to draw on world knowledge than viewers, and their recall of correct information was worse, sometimes because they substituted real-world knowledge. They recalled fewer precise and vivid details. The authors also reported, though this was not measured systematically, that readers showed little emotion, whereas at least two film viewers cried and some film groups applauded (1979). As the authors point out, this potential difference in emotion may have had an impact on memory. However, this need not indicate a general distinction between media: texts have the potential to generate emotion too. In this respect, the experiment illustrates rather how equivalence on one dimension (structure or content) inevitably interferes with other dimensions (doctoring the text to match structure or content can hamper the stylistic freedom that might make it more emotional). It is not clear then how far Baggett’s findings relate to differences in psychological effect of media in general rather than differences between, for example, the comparative emotional power of these specific stimuli.

A second body of work contrasting moving image and verbal narrative arose from growing concerns in the 1970s about the rise of television and its power to reduce time spent by children reading (Neuman, 1995). Findings here are hard to compare because of varied uses of stimuli across moving images, audio, text, and still images, alone or in a range of combinations, and usually by young children, since researchers are usually interested in issues relating to children’s competence in literacy (Singer and Singer, 1981, 2005; Beagles-Roos and Gat, 1983; Pezdek and Stevens, 1984; Greenfield et al., 1986; Pezdek et al., 1987; Beentjes and van der Voort, 1988, 1991; Gibbons et al., 1991). With these caveats in place, some findings are consistent with Baggett’s. Beagles-Roos and Gat, for example, found that (child) readers are more likely to use real-world knowledge to form narrative inferences than viewers; similarly, Baggett found that (adult) readers were more likely to have real-world knowledge intrude into recall protocols (Beagles-Roos and Gat, 1983). Gibbons et al., as well as Beagles-Roos and Gat, found, like Baggett, that viewers had a better memory for content in general and detail in particular than readers (Gibbons et al., 1991). Levorato (1991), on the other hand, found that children who watched a video of a familiar line of action (getting ready for bed, laying the table) recalled minor actions in the sequence less well than those who read a verbal description and that the readers produced more linguistically complex reports than the video group.

Existing research also suggests that differences in processing verbal narrative may vary between individuals and groups, in ways that are likely to affect a comparison with a moving image narrative. Mental imagery abilities, for example, vary in vividness when measured by questionnaires such as the Vividness of Visual Imagery Questionnaire (Marks, 1973; Zwaan, 2009; for a critique of the questionnaire, see Troscianko, 2013). Brain imaging and clinical research also point to high levels of individual variation in imagery, with the size of area V1 both “predicting the sensory strength and precision of visual imagery” and “likely to vary enormously across individuals” (Pearson et al., 2015, p. 594). Denis (1982) found that high imagers have better recall for narrative and descriptive text than low imagers, an advantage which disappears for abstract non-imageable texts. Center et al. (1999) found that imagery training improved comprehension and recall for written narratives in children. Weibel et al. (2011) found that imagery abilities are not related to either the presence or enjoyment uniformly across the media of text, film, and computer game. Individuals with high imagery abilities experienced more presence and enjoyment in text, while those with low imagery abilities reported “marginally higher enjoyment ratings” in film, but not higher presence. Individuals with high imagery ability, then, may find text easier and more rewarding than those with low imagery, a factor which will affect their differential response to medium. At least one set of findings, however, suggests limits on the benefits of high imagery for texts. Readers can adjust to some counterfactual norms, such as talking pigs, very rapidly (Foy and Gerrig, 2014), and “minimally counterintuitive concepts,” where a natural law is broken (for example, a talking bush), are found routinely in fairy tales (Thompson, 1955–1958; Norenzayan et al., 2006). It is not clear whether the medium contributes to this easy adjustment, but Slone et al. (2007) find that while high imagery (for example, “apple” versus “justice”) interacts with memory for both intuitive and maximally counterintuitive concepts, this was not the case for minimally counterintuitive concepts. “Unnatural narratologists,” for example, argue that genre expectations create new norms, so that minimally counterintuitive concepts are not just accepted, but seen as natural for a reader/hearer of fairy tales (though see also Alber, 2016; Anderson and Iverson, 2018; Jajdelska, 2019).

As well as differences between individuals in imagery, there is evidence of differences between individuals in speeds of visual and verbal processing. These can be detected in infancy: verbal and spatial working memory capacities have high heritability. These individual differences interact with affect in ways that lead to “preferred brain pathways to process visual and cognitive information” (Parasuraman and Jiang, 2012, p. 77). Traits such as “high need for cognition” may also differentiate the text and movie experiences of individuals. Allbritton and Gerrig suggest that reading narratives will be more transportive than viewing them (Allbritton and Gerrig, 1991). But Green et al. (2008) found that while imagery was not related to transportation, high need for cognition individuals found text narrative more transportive, while the converse was true for low need for cognition individuals. The personality trait of openness has also been associated with a preference for print fiction, again suggesting a difference between the ease and pleasure with which individuals might process verbal and film narrative (Mar et al., 2009). Traits such as anxiety can affect readers’ predictive inferences in relation to threat. More anxious readers are more likely than less anxious ones to make predictive inferences in response to threat-based content (Calvo and Castillo, 2001). It is not clear if these individual variations are equally relevant to the experience of moving image narrative. Finally, variation in verbal competence (written and spoken) plays an obvious role in differentiating between reader experiences compared to those of viewers (Van der Molen, 2000).

Age too can affect narrative processing by medium (Hayes and Birnbaum, 1980; Baggett and Ehrenfeucht, 1982; Bohn-Gettler et al., 2011). There is a marked difference between children of around 4 years old and those of 8 or 10 years, with younger children in particular performing better on movies than text on recall and comprehension (Reifel, 1984; Gibbons et al., 1986; Beentjes and van der Voort, 1991; Bordeaux and Lange, 1991; Kendeou et al., 2008). Younger children (about 4 years old) tend to infer goals from explicit physical actions more than other sources of information. This may explain why they recall audiovisual narratives better than audio ones, whereas the older children and adults in the same studies, conducted over generations with different levels of exposure to visual media, tended to perform equally well in both media (Hayes and Birnbaum, 1980; Baggett and Ehrenfeucht, 1982; Reifel, 1984; Gibbons et al., 1986; Beentjes and van der Voort, 1991; Kendeou et al., 2008). As children get older, they get better at narrative processing in both media (Pezdek et al., 1987). But although, as we saw above, both verbal and moving image narrative comprehension require skill and practice, verbal narrative comprehension in particular seems to benefit from increased practice in childhood (Beentjes and van der Voort, 1988; McGeown et al., 2015; Richie et al., 2015; Jensen et al., 2016). Differences between children of the same age in processing narrative relative to medium may reflect different levels of practice as well as differences such as need for cognition or imagery ability.

As well as these differences between individual experiences of the two media, Magliano et al. (2013) propose structural differences. “Shots,” they argue, can be compared to “sentences” as “minimum units of production.” However, it can be argued that shots can just as plausibly be compared to words. And there are problems with either analogy. In McQueen and Walsh (2008), for example, a single 17-and-a-half-min shot covers an unbroken dialogue between two characters (Maher, 2008). In ecological settings, film narrative often requires visual, verbal, and musical processing simultaneously. Silent readers control the pace of the narrative; solitary viewers can rewind or fast forward, but cannot otherwise control pace, and film watching is often social (Magliano et al., 2013, p. 80–82). The power of film to control visual attention through shot length, motion, luminescence, and other medium specific affordances has been described as “the tyranny of film” (Hasson et al., 2008; Cutting et al., 2011; Loschky et al., 2015). This characterization has some support from some evidence of synchronization of eye blinks and saccades between viewers of film (Nakano et al., 2009; Shimamura et al., 2014; Loschky et al., 2015). However, this degree of synchronization between viewers may not apply to longer-term processes, such as identifying characters’ goals (Kauppi et al., 2010). There may also be a bias here to mainstream commercial film and a potentially reductive model of passive viewer and active filmmaker. A comparison between *auteur* film and *commercial* fiction books, or silent film highly formulaic genres, might instead highlight text’s power to control attention through, for example, “rhetorical focusing,” and film makers’ power to make viewers work hard through choice of cuts or camera angle (Schwan and Garsoffky, 2004; Sanford and Emmott, 2012).

## Summary: Similarities and Differences

There are considerable overlaps in the affordances of different kinds of narrative media, and these are reflected in processing. Viewers and readers alike can be constrained by the attention hierarchies of real life when they segment narratives into events using situation modeling. These sequences of events allow readers and viewers to retell the story as a new narrative in the original or a different medium, explaining, for example, why novels can be turned into recognizable films. Both media require viewers or readers to see the narrative as an artifact with the hand of a human maker behind it in order to comprehend it, and this can involve learning conventions of form. Each also has a body of formal techniques that can exploit their different affordances, varying *how* the story is told. At least some of these have comparators in the other medium, allowing for some equivalent or near equivalent esthetic effects. They can both produce empathy, transportation, vividness, and emotion.

**Figure 1:**
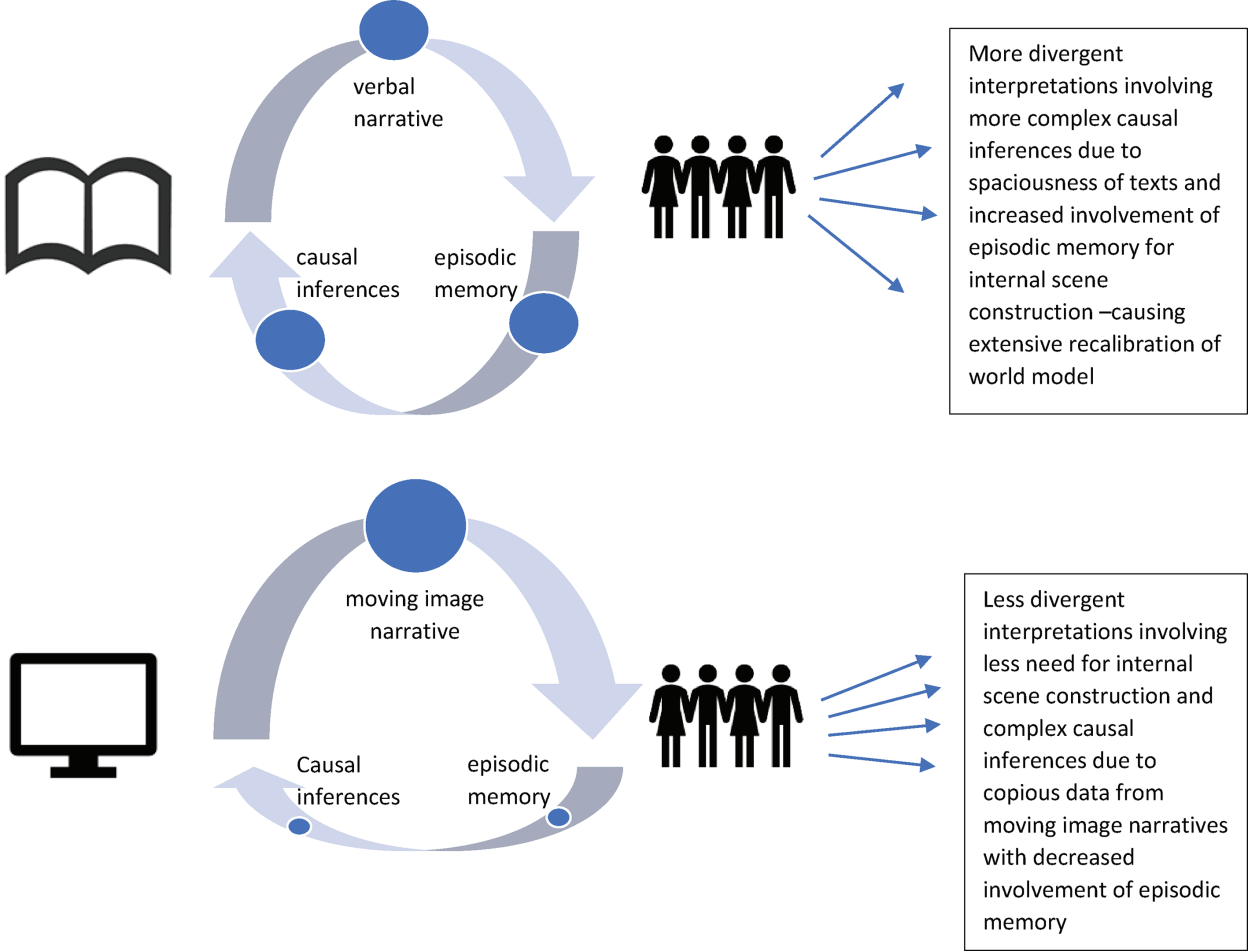
Summary comparison of verbal and moving image narrative processing.

Differences between the two processing experiences may be manifest in the greater variety among readers’ memories of the story, as they draw more heavily on their own experience of the world to form inferences (see Figure 1). In film groups, for example, the role of negotiating the self when reconstructing a story can be made open, allowing for high mutual influence in self-presentation to the group (Edwards and Middleton, 1986). In book groups, the process of jointly remembering and interpreting the text may involve both synchronization of mood and speech and divergence in interpretation and recall of the text (Steenberg et al., 2014). Readers’ memories may be not only less accurate than those of film viewers but also more creative in the sense of introducing new elements. Readers may be less likely than viewers to recall, or believe that they recall, vivid (in the sense of sensory) details. Their retellings may themselves have narrative form; recall of movie stories may be closer to reportage. Readers may also diverge more in retelling because of individual differences between them, including differences between infants, older children, and adults; traits like openness and need for cognition; and aptitudes in mental imagery. It should be noted, however, that all of these distinctions may be affected by both experimental and ecological task demands. A student preparing to answer questions on a narrative in a language exam may remember different details from someone reading, hearing, or viewing for leisure, for example. We also touch on the difficulties of creating equivalent task demands in relation to different media toward the end of this review.

## Non-Narrative Visual and Verbal Processing

The body of work directly comparing film and text narratives is small, but work on other aspects of verbal and visual processing may also be relevant. Some aspects of visual processing may be “cost free” in comparison to their verbal equivalents (Lang et al., 1995 citing: Salomon, 1984; Salomon and Leigh, 1984; Graber, 1990; Grimes, 1991; Rolandelli et al., 1991; Basil, 1995). For example, visual perception of physical causation can be an automatic process, involving fine grained spatial and temporal congruity that verbal description cannot reproduce (Fugelsang et al., 2005). Similarly, visual processing of human action can engage automatic simulation at fine grained levels (Brass et al., 2000).

Moving images therefore might be expected to have an advantage over text in teaching new tasks. The evidence here is hard to interpret. Studies of learning to use a new computer application (Palmiter et al., 1999) or an asthma inhaler (Wilson et al., 2010) indicate an advantage for video in short-term recall (Wilson et al., 2010) and task performance (Palmiter et al., 1991). But this advantage may be limited to participants with lower literacy skills (Wilson et al., 2010), or reversed in longer-term task performance, where viewers were slower and less accurate in a related task (Palmiter et al). Lower competence in literacy is also associated with the advantage of moving image news plus words over still image news in Van der Molen (2000), where children perform better with television and adults with print. Moving image information may involve greater engagement, sympathy, and perceived realism but not necessarily improved processing or recall (Yadav et al., 2011).

Another example of automaticity in visual processing relates to “theory of mind” or “mirroring” interpretations of agents’ goals (perception of actions *via* the mirror neuron system). This can be contrasted with “mentalizing” systems used for the same purpose (Overwalle and Baetens, 2009). The two have different implications for readers’/viewers’ narrative emotions and interpretation. Identification of outgroups, for example, may recruit mirror neuron system responses alone and even categorize these agents or characters as “infra-human” objects (Harris and Fiske, 2006). Verbal descriptions of actions can also engage the mirror neuron system, but much depends on exactly how the passage might be written (Gallagher et al., 2000; Hauk et al., 2004; Jajdelska et al., 2010). Again, careful attention to stimuli is needed.

A long line of research on the “picture superiority effect” (Paivio, 1971) investigates whether pictures of objects have greater effects than single-word depictions of those objects (any effects may extend to auditory representations of the objects; Crutcher and Beer, 2011). Insofar as there is a picture superiority effect, it need not be because of Paivio’s “dual coding” account of processing (Hockley, 2008). However, it is not clear that pictures really do have a general advantage over words in relation to either recall (Bartlett et al., 1980; Gati and Tversky, 1987; Koehler et al., 2005; Reinwein, 2012) or emotion (Otgaar et al., 2010; Schlochtermeier et al., 2013).

The “verbal overshadowing effect” is associated with evidence that describing a face after seeing it can make it harder to recognize that face again (Klatzky et al., 1982; Schooler and Engstler-Schooler, 1990). One possible explanation for this is that face perception is holistic, whereas verbal descriptions tend to be feature by feature (Dodson et al., 1997). However, when verbal descriptions are holistic and less feature-based, they may sometimes improve recognition (Brown and Lloyd-Jones, 2006). In the case of dynamic scenes, rather than faces, Huff and Schwan (2008) find that verbalization (in this case in the form of reading a description rather than generating one) can involve both facilitation and overshadowing. Jajdelska et al. (2010) suggest that differences in the way a text describes a face may significantly improve memory for the described face.

An automaticity advantage of moving image may be plausible in some cases, such as direct perception of causation and action. In case of the picture superiority effects, and verbal overshadowing effects, much may depend on the precise words and images used. Again, this suggests that great care is needed in choice of stimuli when attempting to compare narrative media.

## Interactions Between Text and Image

This is an area that has been researched by educational psychologists. Mayer’s “multimedia principle,” for example, states that “People learn more deeply from words and pictures than from words alone” (Mayer, 2014, p. 43). Again, research on illustrated texts suggests a complex range of interacting effects between visual and verbal processing, often depending on the precise words and images used. In a review of research on how the sequencing of picture and texts relates to learning, for example, Eitel and Scheiter conclude that “the relative complexity of the information conveyed by the picture and by the text should determine which medium is better to be processed first” (Eitel and Scheiter, 2015). In some books, the illustrations and texts jointly supply the information needed, rather than one supporting the other (Corrigan and Surber, 2010). Elsewhere they aid comprehension in relation to some kinds of text, but not others (Moore and Skinner, 1985; Beveridge and Griffiths, 1987; Trabasso and Nickels, 1992). As with the picture superiority and verbal overshadowing effects, small variations in the fine detail of stimuli can have powerful impacts (Schlochtermeier et al., 2013). Magliano et al. (2012), for example, speculate that an illustration of a leaping frog in a children’s book will be more vivid than the accompanying text but do not explore the potential effects of a different text using, for example, kinesic imagery (Jajdelska et al., 2010).

Very few films in recent decades do not use language (through dialogue and sometimes onscreen text and voiceover) and music as well as images. Magliano et al. found that viewers of a popular action film relied on visual sources, music, and dialogue in that order to make predictive inferences (Magliano et al., 1996; Hoeckner et al., 2011; Strick et al., 2015). However, as the discussion of cinematic form earlier suggests, a different film genre might produce different interactions between these three elements. Work on graphic novels and comic books shows that formal features of image and text, such as viewpoint, can conflict as well as harmonize, supplying these media with a particularly rich and challenging range of affordances (Kukkonen, 2011, 2013; Sabeti, 2012). As with research on visual versus verbal processing, research on mixed media suggests that great care is needed if comparing narrative stimuli by medium.

## Issues with Methods Used in Existing Research

Verbal processing, like mental imagery, is increasingly understood as engaging the resources of higher level visual processing (Barsalou et al., 2003; Hauk et al., 2004; Kosslyn et al., 2005; Reddy et al., 2010; Hauk and Tschentscher, 2013; Pulvermüller, 2013). Yet, as we have seen, establishing equivalent stimuli across words and images may be an impossible task. Episodic structure and duration, for example, cannot be made identical without varying style, imagery, and form. Creating equivalent content again may be impossible; just one still from a movie can contain more visual information than the lengthiest description could fully capture. And although film and text share some formal affordances, these cannot often, if at all, be equated in fine detail. Again, it is problematic to vary dimensions one at a time, since each dimension potentially affects the others. The emotional effects of a story, for example, can potentially relate to interactions between all of these dimensions, and emotion cannot necessarily be untangled from perception and comprehension (Calvo and Castillo, 2001; Barrett and Bar, 2009).

Task demands in relation to the two media also present a problem. Verbal narrative is embedded in social life and performance, from everyday conversational narrative to high culture. Sociolinguists, for example, have shown how conversational narrative acts as a means to negotiate self-identity in relation to group identity, while anthropologists have discussed the role of performance in narrative production and audience reception (Labov and Waletzky, 1967; Bauman, 1986; Barber, 2007). On the other hand, few, if any, of us are “everyday storytellers” in film, despite the rapid increase of filmed material on smartphones. Baggett noted that her reading participants retold stories using “Once upon a time” and reproducing past tenses from the original texts, while the viewers reported what happens “when the movie begins” in the present tense (1979). Readers may experience “an illusion of truth” when reading fiction as they have a default position of trusting narrators, a trust harder to replicate in films even if they use a voiceover narrator (Gilbert, 1991; Prentice and Gerrig, 1999; Marsh et al., 2003), although some forms of fiction rely on readers’ distrust of “unreliable narrators.” The processing of verbal narrative, then, even in written form, is embedded in processes of social interaction, and readers/hearers also have a lifetime of experience as narrators of conversational narratives themselves. Recent directions in predictive language processing also emphasize the closeness of production and comprehension, as readers/hearers simulate the speech of interlocutors in order to time their own contributions to conversation (Pickering and Garrod, 2013; compare Overy and Molnar-Szakacs, 2009 on music). Visual narrative processing may involve the simulation of content but is less likely to involve simulation of production, if at all.

Equivalence of stimuli and tasks, then, may be an impossible goal. A possible alternative is to investigate graded effects on a continuum of stimuli. In an analysis of an oral storyteller’s performance, Lwin (2010) shows how the combined effects of the performer’s motion and prosody create a unified interpretation of the text encouraging “relatively uniform cognitive, emotive and evaluative responses” in the live audience. A continuum from silent reading, to audio only, to live performance with different degrees of motion could then be used to grade how far audience responses diverge from one another at each stage. Similarly, experiments on serially degraded images could be extended to moving images to identify the relationship between viewer experience and patterns of withheld information (Churchland, 2012, p. 66–67).

An additional approach is to pursue a clearer understanding of low-level processing mechanisms; “further research on the effects of back-end processes on front-end processes across media is greatly needed” (Magliano et al., 2013, p. 88). In what follows, we identify internal scene construction and visual/verbal working memory as promising areas for future research.

## Future Directions: Internal Scene Construction and the Episodic Memory System

It is now understood that the episodic memory system is used in a range of tasks beyond event memory, including future planning and imagination (Hassabis and Maguire, 2007; Zeidman et al., 2015). There is also a relationship between episodic memory, the self, and narrative production. This can be seen in the co-emergence in childhood of narrative and episodic memories (Nelson and Fivush, 2004; Hoerl, 2007), the pressure on autobiographical memories to cohere with beliefs about the self (Conway, 2005), and the role of memory in developing a narrative of the self (Fitzgerald, 1988; Wang and Conway, 2006). This relationship may also be seen in findings on the relationships between personal experience and narrative processing (Chow et al., 2015), on false memories in pictures versus verbal narratives (Garry and Wade, 2005), and on a “self-reference” effect (Carson et al., 2016). The default mode network may moderate this relationship between episodic memory, narrative, and the self, through a role in generating narrative both during task-related activities and in “resting” or “screen-saver mode” (Gerrans, 2014, p. 5). Pearson et al. (2015) relate the physical closeness of “high level areas … to memory-encoding structures” to the overlap in perception and imagery, and the claim that “mental imagery is presumably based on the recall and recombination of memories” (595). It is perhaps unsurprising, then, that readers can engage in episodic future thinking in which they project their selves on to characters (Buckner and Carroll, 2006; Finnigan, 2013, p. 151–160).

Experience of verbal narratives, rather than moving image ones, may supply models for the social presentation of the self through autobiographical narrative (Labov and Waletzky, 1967; Habermas and Paha, 2001; Rubin et al., 2011; Jobson et al., 2014). Fioretti and Smorti (2015) compared autobiographical reminiscences with narratives of those memories. The narratives included more emotion and emotional complexity than the reminiscences, especially in relation to surprise, recalling Bauman’s account of narration of stories creating more suspense when performed orally to unfamiliar audiences (Bauman, 1986).

Neuropsychological evidence suggests that a crucial element in this set of relationships between narrative, episodic memory, and the self is the ability to imagine scenes (Hassabis et al., 2007). Damage to the medial temporal lobe (MTL) affects both episodic memories for one’s own past and the ability to imagine the future (Tulving, 1985; Addis et al., 2007; Andelman et al., 2010). Race et al. (2011) asked patients with MTL lesions to: remember specific personal events from the past; imagine specific personal events in the future; and imagine that each of five detailed drawings of a scene, shown sequentially, was a scene from a movie and to tell a story about the scene. As in previous studies, both episodic memories and future thoughts were significantly less detailed than those of controls. But this was not the case for the pictures, which acted as an external memory of the relevant scenes to afford narrative production (a role which illustrations may sometimes play in relation to text; Glenberg and Langston, 1992; Kruley et al., 1994). The capacity to generate internal scenes, then, is important in remembering, imagining, and producing narrative.

Verfaellie et al. (2014) worked with eight patients without the pictures of scenes used in Race et al. (2011). Researchers selected five fairy tales and four Bible stories. MTL patients were impaired in their ability to recount detailed semantic narratives, despite retaining narrative structure and recognizing story details. The loss appeared to be not of semantic knowledge but of the ability to recollect it in rich detail. This again suggests that the patients in Race et al. (2011) used the drawings of scenes for external scene construction in order to generate a richer narrative, and that the narrative obstacle experienced by the patients in Verfaellie et al. was the inability to imagine a scene in detail. As patients in Mullally et al. put it:

it’s as if I have a lot of clothes to hang up in a wardrobe, but there’s nothing to hang them on, so they all fall on the floor in a complete mess. (266)

I’m imagining different things happening, but there’s no visual scene opening out in front of me. (266)

It’s hard trying to get the space. It keeps getting squashed. (266) (Mullally et al., 2012)

Internal scene construction, then, appears to be an important element in processing verbal narrative. And the findings in Race et al. suggest that film viewers may be spared some or all of this cognitive effort. Readers and hearers with impaired scene construction abilities can, to varying degrees, recall story, the bare sequence of events associated with event perception and identified by anthropologists and narratologists as a vehicle for richer retellings. But they struggle to go beyond this.

The role of internal scene construction in verbal narrative may go beyond merely decorating underlying stories with visual detail. Ahmed et al. find that patients with Posterior Cortical Atrophy, who have damage to the visual cortex, perform poorly on tests of autobiographical recall. This is not because there is less overall information in their narratives but because their narratives lack visual and perceptual detail. This seems to be replaced by “semantic” detail, detail external to the event itself, which is included in an explanatory capacity (Ahmed et al., 2018). In the narratives of patients with mild Alzheimer’s disease or amnesia compared with those of healthy participants, the loss extends to character motivations, the agents’ goals which lie at the heart of situation modeling and earlier “story grammar” approaches to narrative structure (Addis et al., 2009; Schacter et al., 2013; Verfaellie, 2014). This leaves a temporal sequence of actions with under- or unspecified causal relations to one another, potentially leaving patients to rely on serial memory for recall (Gentner, 1976). This kind of skeletal sequence of events, referred to in this review as “story,” with absent or ambiguous causation between episodes, has been identified by folklorists analyzing the underlying structure in variants of oral narratives such as folk and fairy tales (Aarne and Thompson, 1961; Bauman, 1986). It also recalls Bartlett’s findings, in which English participants retold a native American tale with new, more explicit character motivations, without realizing they had done so (Bartlett, 2014 [1932]). The different versions of a tale type can indeed vary dramatically from one another in causal links between episodes, especially when they cross cultures. A villain in one version of a tale can even become a victim in another version (Trompf et al., 1988). The transmission of folk tales, therefore, suggests that a story, or bare sequence of events, and the causal structure relating those events in a particular narrative telling, are separable. Narrative recall by patients supports this suggestion that episode sequence and causal relationships are separable. Moya et al., for example, found that right hemisphere damaged patients “were impaired on all aspects of visuospatial performance and verbal recall” of their narratives. These patients had difficulty in “interrelating components to one another, drawing inferences, and selecting an appropriate structure” (Moya et al., 1986, p. 387).

Internally constructed scenes, then, may be more than just a vehicle for vivid detail; they may build in the structures of causation missing from some patients’ narrative retellings. Research on boundary extension supports this claim. Boundary extension is an error in which scenes are remembered as extending further than they actually do (Intraub and Richardson, 1989):

When we initially encounter a scene, we are not limited to the information that is in front of our eyes, but have access to an automatically constructed and implicitly maintained internal representation of the scene…[which] extends well beyond the borders of the given scene and provides an overarching framework into which we rapidly embed what is currently in our field of view. This is a highly adaptive process that supports our experience of a continuous and coherent world, despite it being amassed from discontinuous sensory input. (Mullally et al., 2012, p. 261)

Patients with hippocampal lesions show attenuated boundary extension; the patients remember scene boundaries more accurately than controls *because* they appear to “have a fundamental problem generating internalized scene representations” (263). As with Verfaellie et al. (2014), patients did not have difficulties in perceiving the pictures of scenes. With scenes before them, they could also “anticipate what might be beyond the view in the scene” (263). However, when asked to imagine an extension of the scene, while patients could predict what might be there, their descriptions lacked spatial coherence. They could bring to mind contextual associations but not organize them in spatial relationships (266).

Spatial relationships are integral to causal relationships (Brass et al., 2000; Fugelsang et al., 2005). Even in a static scene, spatial information lets us calculate a range of potential causal interactions in the future. Simply knowing that a scene has a plant pot, a person, and a watering can in it, without seeing that scene, already raises the possibility of the person watering the plants. But if we see that the plant and the can are both situated on a high shelf above the person’s sight line, then we can model more accurately the agent’s likely patterns of attention and behavior, the physical effort required to take down plant and can and so on, and our range of potential causal interactions is both expanded and refined. “Causality,” in this sense of forces in a space, need not be distinguished from the character goals, motivations, and interactions with objects identified in situation model theory (Zwaan and Radvansky, 1998). There is a certain probability that the person will reach for the watering can, another that the can will fall by accident on the person’s head alongside all the other potential options, including no interaction with can or plant at all. Both kinds of cause (pursuit of agents’ goals and the laws of physics) are built into the scene’s construction and potentially interact with one another.

Readers then, compared to viewers of film, may rely much more heavily on *internal* scene construction not just for ornamental vivid detail but for a unified sense of the set of possible interactions between all the elements of a scene, from agents and objects to surfaces and volumes, and therefore for local and global causal (and not just serial) narrative connections. Since the internal scenes generated by verbal narrative are likely to be more sparse than those generated by an ongoing film narrative, the causal relations between the elements of those sparser scenes are also likely to be more flexible and to have more variation between individuals. A film, for example, can capture fixed and precise relations of shadow and proximity, with causal implications for the way elements in the scene, including people, relate to one another. This suggestion is consistent with current views of concepts as “flexible, distributed representations comprised of modality specific conceptual features” (Kiefer and Pulvermüller, 2012, p. 805). Readers have more scope to exploit this flexibility than viewers as they instantiate concepts through scene construction, with ensuing flexibility in generating causal explanations at local and global levels (Jajdelska, 2016).

In this way, narrative causality may be more highly determined between individuals by film than by text. As Heider (1944) observed, “a change in the environment gains its meaning from the source to which it is attributed. This causal integration is of major importance in the organization of the social field” (372). Character traits and agency, key to narrative processing, may seem intuitively to be comprehensible without spatial representations. But Heider and Simmel (1944) show how pregnant with agent-directed change a spatial scene can be. Similarly, Finnigan observes that verbal descriptions often mimic the holistic early stages of visual scene perception (Finnigan, 2013), and Fugelsang observes that “extracting causal structure” is “an inherent property of the visual system” (Fugelsang et al., 2005, p. 45). Findings by Jahn (2004) also suggest that causal relevance is “a precondition for the spontaneous construction of spatial situation models” (see also Radvansky and Copeland, 2000). Even those narrative causal relations that seem to go beyond the direct phenomenal causality implied in a spatial representation, those which are often thought to require reasoning (“It was probably the drink because he fell in love while drinking the cocktail”), can be related to a spatial framework (Oestermeier and Hesse, 2000). Kruley et al. (1994) suggest that spatial representations are used to represent non-spatial information. Negative and positive emotions, for example, can be mapped in contrasting areas of space (Myachykov et al., 2017). Internal scene construction, then, suggests how early processing can be related to higher level of interpretation over shorter or longer time scales, through support for actual and potential causal relations at different levels of abstraction. Indeed, visual scenes may just be a prominent example of a wider family of complex inter-relations between “objects” necessary in imagination. There is work, for example, on “auditory scenes,” which have a comparable relational structure, and the perception/imagery of which is also impaired in hippocampal patients (Teki et al., 2012). Social hierarchies also rely on comparable relational structures (Kumaran et al., 2012).

Their higher reliance on internal scene construction, then, suggests that readers will generate a more varied and flexible causal model to underpin the narrative than viewers. In addition, the sparser visual information of internally constructed scenes can allow for more flexible and individualized instantiation of concepts than that of externally visible moving images (Mahon and Caramazza, 2009; Kiefer and Pulvermüller, 2012). These differences may afford readers a closer relationship between the self and narrative processing than viewing does, as readers draw more heavily on memories of their own lives to construct internal scenes. Gordon et al. (2009), for example, found that memories for text narratives resembled memories of imagined events, whereas memories for film narratives resembled memories for real-world events. Garry and Wade (2005) found that readers of modified personal text narratives were more vulnerable to false memories than those who saw doctored photographs. Carson et al. (2016) find a “self-reference effect” for text narrative, where events related to the self are better remembered than those which are not.

Neuropsychological evidence, then, will likely prove important to the future of narrative research by medium, as will methods of measuring how far memory, imagination, and navigation systems are engaged, for example, through measures of glucose consumption in retrosplenial cortex (Vann et al., 2009).

## Further Directions for Future Research: Working Memory, Predictive Processing, and AI

We saw earlier that individual differences in imagery may interact with narrative medium. There is some evidence that high imagers also differ from low imagers in strategies for working memory tasks. Pearson et al. (2015), in a review of mental imaging research, find differences between higher and lower imagers in the strategies used for visual working memory tasks (594; see also Smith et al., 1996). Low imagers pick out details from a scene or array, encode them phonologically, and then compare to the subsequent stimulus. High imagers create a mental image and compare it directly with the stimulus (Gur and Hilgard, 1975; Berger and Gaunitz, 1979; Harrison and Tong, 2009; Keogh and Pearson, 2014; Magliano et al., 2016). This suggests that the possibility of a set of interactions between the episodic buffer; the default mode network (associated with combining meanings to form narratives); episodic long-term memory; and the two “slave systems” of working memory (the phonological loop and the visuospatial sketchpad (Baddeley, 2008, 2018; Smallwood et al., 2016, p. 326, citing Olson et al., 2007; Domhoff, 2018, p. 154–161). This in turn suggests a potential pathway from local, short-term narrative inferencing mechanisms to larger narrative meanings and structures. Differential strategies at the working memory level between high and low imagers may offer a way to explore how that pathway is affected by narrative medium.

New theoretical frameworks for cognition as predictive, and involving high-dimensional vector spaces, may also suggest new methods to capture low-level narrative inferencing mechanisms. Spivey (2007), for example, has drawn attention to increasingly influential Bayesian models of the brain, continually generating and revising predictive hypotheses by drawing on all current input simultaneously, from bottom-up to top-down. Older, modular models of processing, in which one task is completed before the next can be begun, were suited to behavioral experiments in which task outcomes were measured once, at the conclusion of the task. Spivey argues that the Bayesian brain can be better understood by continuously measuring activity throughout the performance of the task. For example, tasks that require participants to choose one of two verbal alternatives to complete a phrase can be measured using the participants’ movements of a computer mouse, which tracks their movements to and from the potential targets leading up to their final decision. Adapting this approach to moving image processing might allow a comparison between low-level mechanisms for narrative processing that did not rely on attempts to produce equivalent stimuli. Emerging neural, or deep learning, AI networks use high dimensional vector spaces to learn processing skills. These are still some way from successfully processing narrative in any medium, but they can potentially be used for proof of concept. Inverting neural networks that give verbal labels to images has revealed that they create some surprising implicit causal relationships in the way they interpret their training data (Mordvintsev et al., 2015). Investigating this implicit causality might shed light on the process of generating causal links between mental images produced in response to verbal input. Here too there may be scope for exploring potential low-level processing differences by medium, which can illuminate internal scene construction by humans.

## Conclusion

Comparing narrative processing by medium is difficult because it is not possible to have equivalent stimuli. To address this problem, we recommend a focus on neural mechanisms at comparatively low levels of processing and time scales, in combination with an awareness of the rich and holistic nature of narrative experience, which can encompass memory, imagination, empathy, spatial resources, inference, emotion, and transportation as well as the potential to create meaning in relation to the self. The wide range of benefits associated with reading or hearing fiction, coupled with the richness of the experience, suggests that a simplistic explanation involving cognitive transfer may not be available (Melby-Lervåg et al., 2016). Existing work points to a greater diversity among readers/hearers of verbal stories than among viewers of moving image stories. We suggest that one explanation for this finding is that processing written fiction relies more heavily on the resources of the episodic memory system. This suggests a mechanism that could help explain the different effects of the two media, with written fiction using internal scene construction to make causal predictions, and in doing so, readers/hearers modify their existing model of theworld more extensively than viewers and more distinctly from one another. Narrative processing, it seems, is more than information processing. Readers and viewers may extract an underlying plot structure from films and books, which share a narrative, but their experiences may nonetheless be very different.

## Author Contributions

EJ contributed to identifying articles, coordination, or reviews; writing; and redrafting the manuscript. All authors have contributed to reading and reviewing the articles and commenting on drafts.

### Conflict of Interest Statement

The authors declare that the research was conducted in the absence of any commercial or financial relationships that could be construed as a potential conflict of interest.
